# Supplementary material for: All-polymer particulate slurry batteries
Source: Nat Commun. 2019 Jun 7;10:2513. doi: 10.1038/s41467-019-10607-0 (PMC6555790; doi:10.1038/s41467-019-10607-0)
Supplement: Supplementary file 2 — Supplementary Information [file 41467_2019_10607_MOESM2_ESM.pdf]

Supplementary Information

All-Polymer Particulate Slurry Batteries

Yan et al.

Supplementary Figures

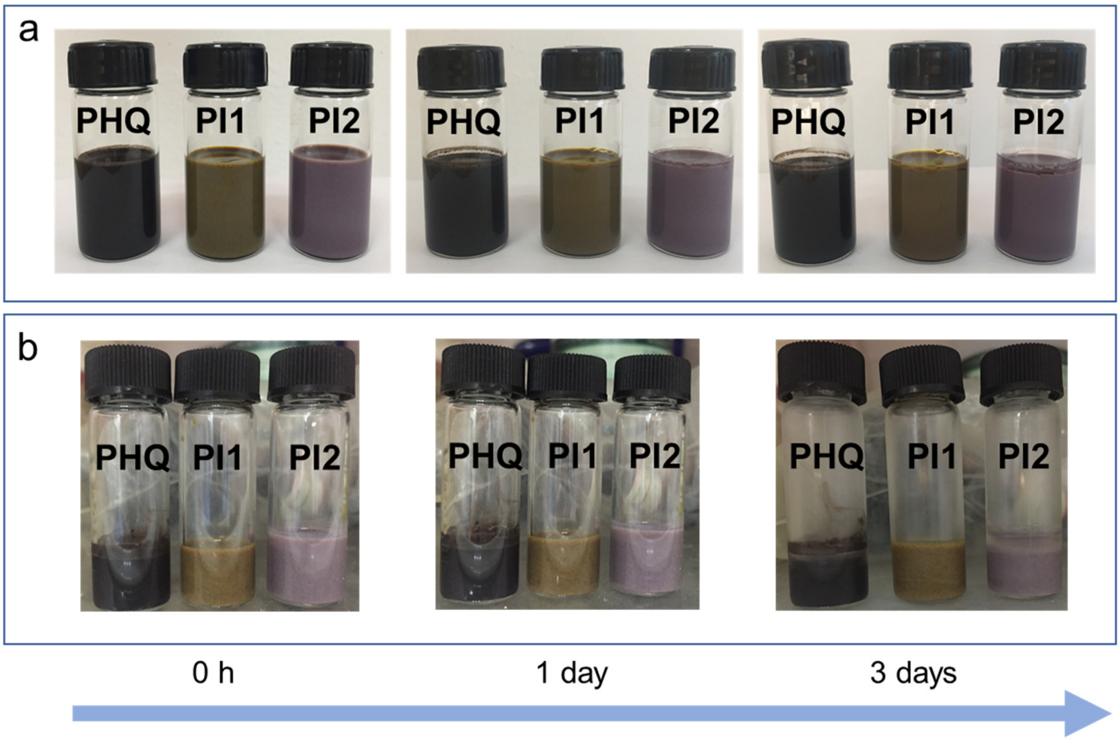

Supplementary Figure 1. Visual observations of the dispersion stability of polymer particulate slurries. Photographs of polymer particulate slurries after different static durations at (**a**) 25 °C and (**b**) 4 °C.

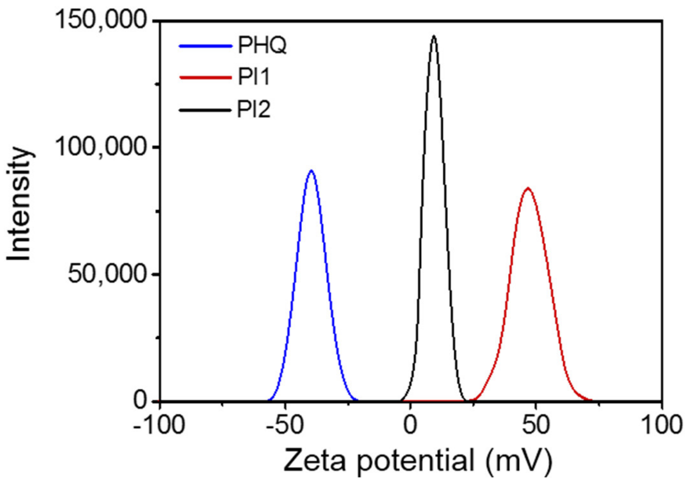

Supplementary Figure 2. Zeta potentials of PHQ, PI1 and PI2 particulate suspensions.

a

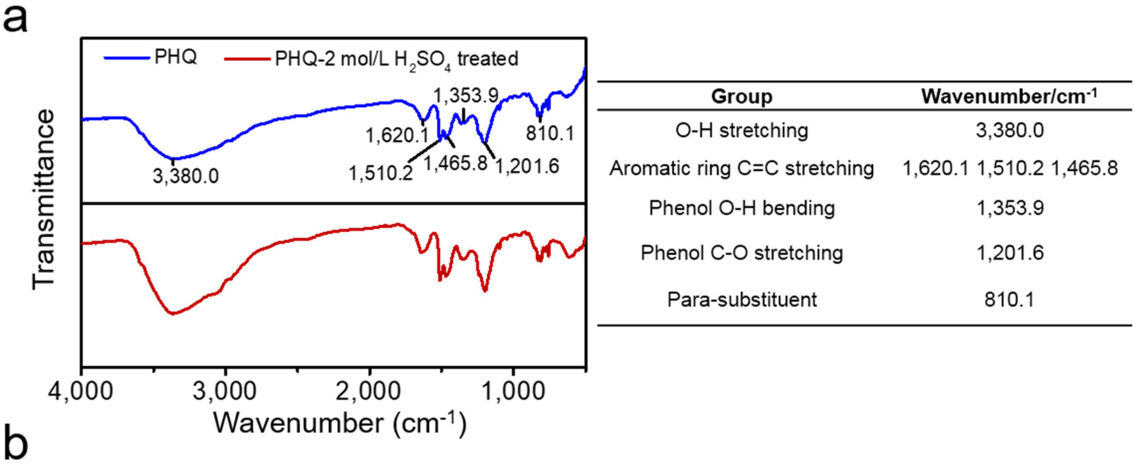

Group	Wavenumber/cm^{-1}
O-H stretching	3,380.0
Aromatic ring C=C stretching	1,620.1 1,510.2 1,465.8
Phenol O-H bending	1,353.9
Phenol C-O stretching	1,201.6
Para-substituent	810.1

b

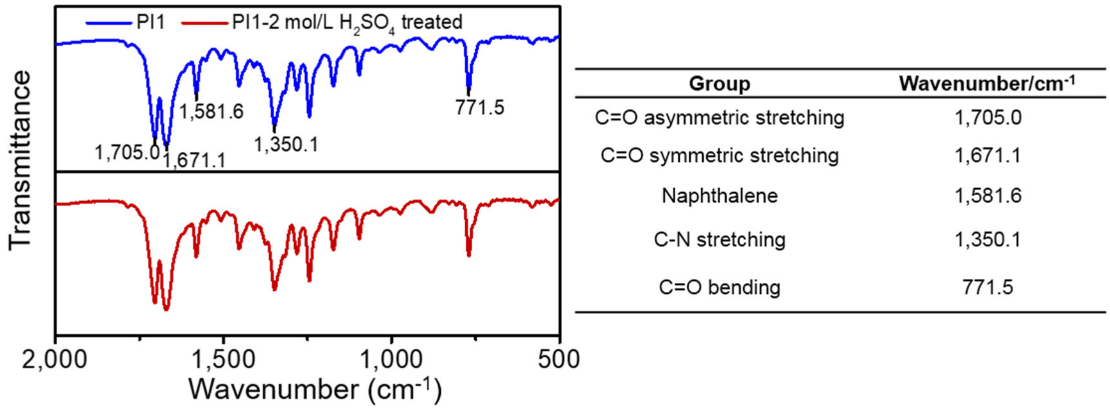

Group	Wavenumber/cm^{-1}
C=O asymmetric stretching	1,705.0
C=O symmetric stretching	1,671.1
Naphthalene	1,581.6
C-N stretching	1,350.1
C=O bending	771.5

c

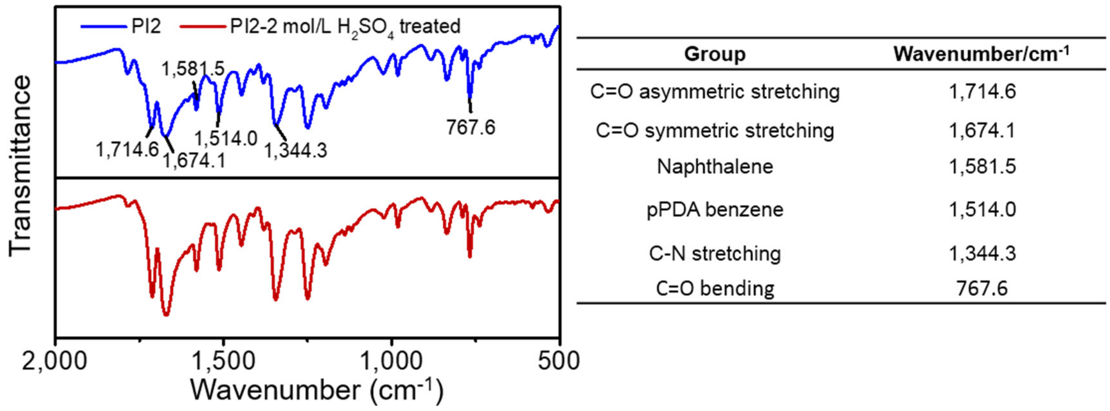

Group	Wavenumber/cm^{-1}
C=O asymmetric stretching	1,714.6
C=O symmetric stretching	1,674.1
Naphthalene	1,581.5
pPDA benzene	1,514.0
C-N stretching	1,344.3
C=O bending	767.6

Supplementary Figure 3. FTIR spectra of PHQ and PI particulates. FTIR spectra of (**a**) PHQ, (**b**) PI1 and (**c**) PI2 particulates, respectively. The characteristic IR band assignments of the three polymer samples are listed on the right.

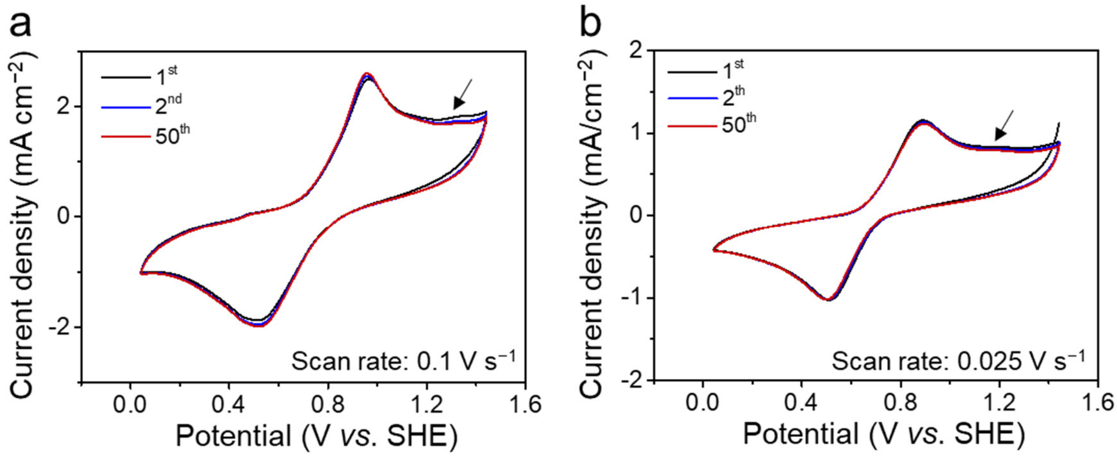

Supplementary Figure 4. CV profiles of PHQ particulate suspension. CV curves of 0.005 mol L^{-1} PHQ particulates in 2.0 mol L^{-1} H_2SO_4 aqueous solution during the 1st, 2nd and 50th cycles at different scan rate of (**a**) 0.1 V s^{-1} and (**b**) 0.025 V s^{-1}, within a potential range from 0 to 1.5 V *vs*. SHE.

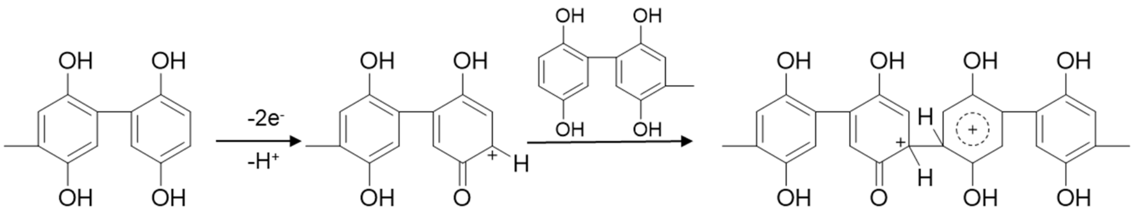

Supplementary Figure 5. Proposed electro-polymerization process of PHQ in acidic solution.

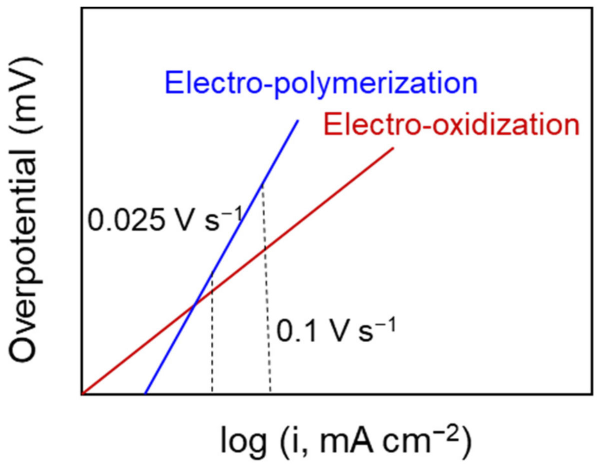

Supplementary Figure 6. Tafel plots of proposed parallel reactions of electro-polymerization and electro-oxidation of PHQ.

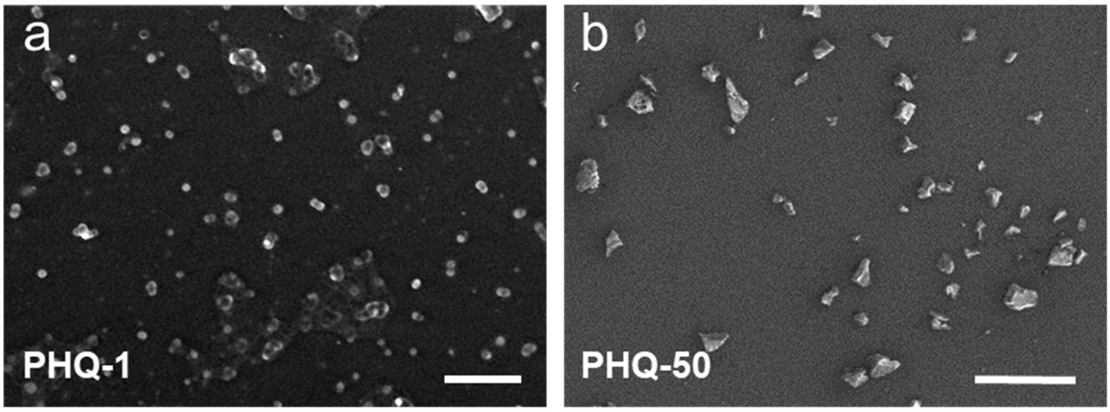

Supplementary Figure 7. SEM characterization of polymer particulates with different sizes. SEM images of (**a**) PHQ-1 and (**b**) PHQ-50. Scale bars: Scale bars, (**a**) 10 μm; (**b**) 200 μm.

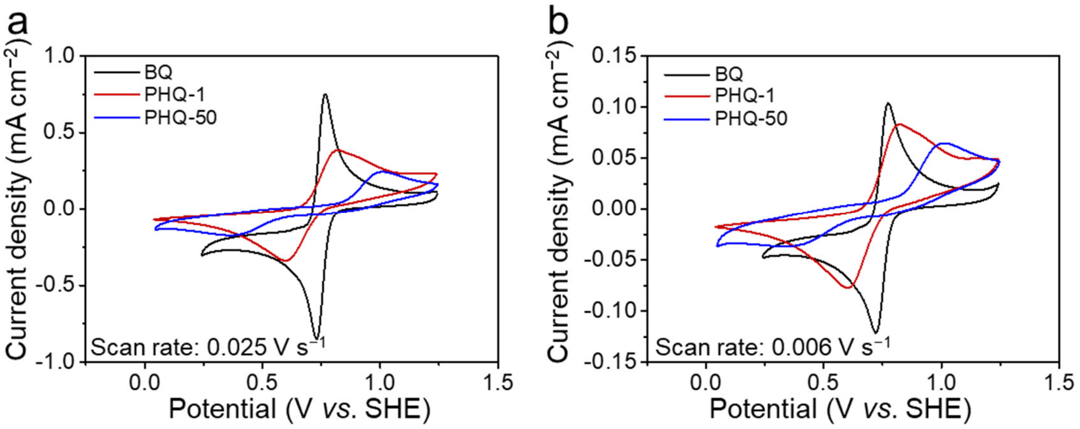

Supplementary Figure 8. CV profiles of BQ and PHQ particulate suspensions with different sizes. CV curves of 0.005 mol L^{-1} BQ, PHQ-1 and PHQ-50 in 2.0 mol L^{-1} H_2SO_4 aqueous solution at the scan rates of (**a**) 0.025 V s^{-1} and (**b**) 0.006 V s^{-1}, respectively.

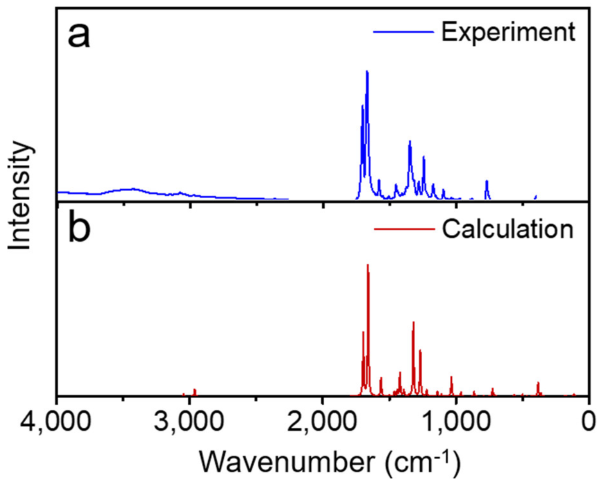

Supplementary Figure 9. Infrared spectra of PI1. (**a**) experimental result (the same FTIR spectrum in Supplementary Figure 3b). (**b**) DFT-calculated result at B3LYP/6-31+G (d, p) level.

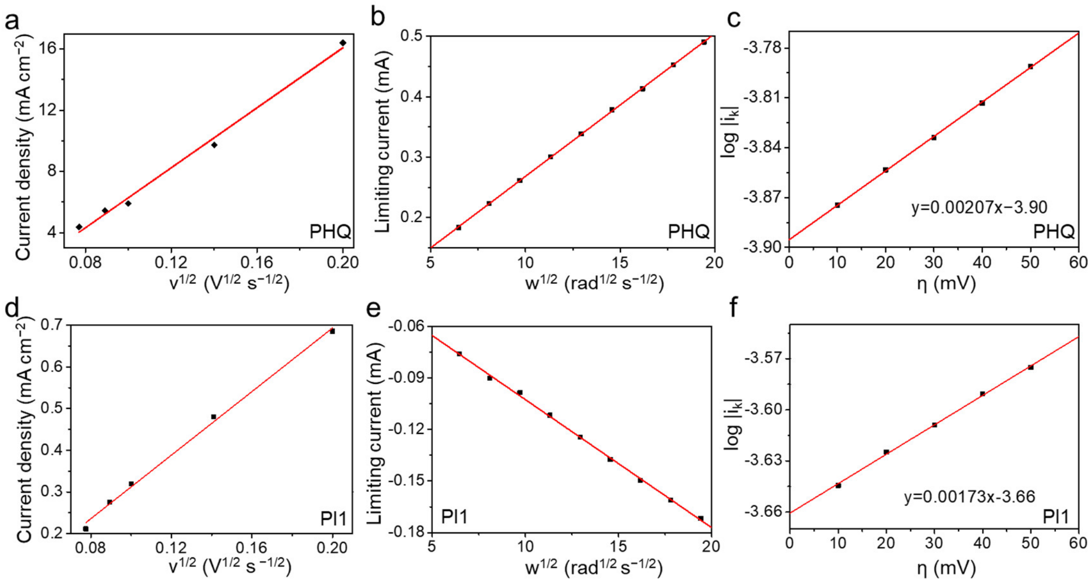

Supplementary Figure 10. Electrochemical analysis of PHQ and PI1 particulate suspensions. (**a**,**d**) Oxidation peak current (i) of PHQ or PI1 particulates versus voltage scanning speed. (**b**,**e**) Limiting current of PHQ or PI1 particulates versus the square root of rotation velocity (Levich-plot). (**c**,**f**) Tafel plot of PHQ or PI1 particulates.

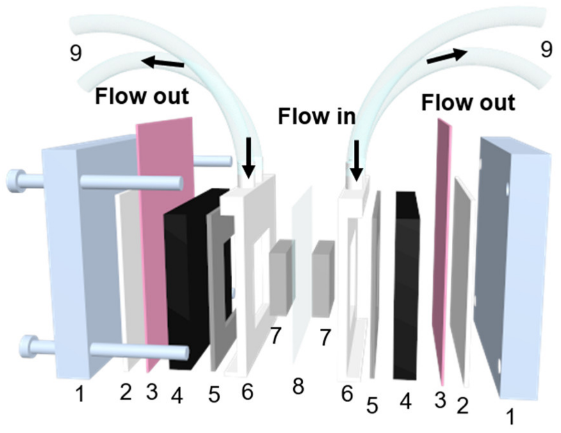

1. Stainless steel plate
2. PTFE plate
3. Titanium current collector
4. Graphite plate
5. Silicone rubber seal
6. PTFE frame
7. Carbon paper electrode
8. Separator
9. Silicone rubber tube

Supplementary Figure 11. Structure configuration and components of APPSB cell system. The stainless-steel plate fixed with screws works as the frame of the cell. The PTFE plate prevents the short-circuit of the cell. The titanium plate acts as a highly corrosion-resistant current collector. The graphite plate with narrow grooves provides the flow channel of the circulating electrolyte, and the carbon paper electrode provides the electrochemical reaction sites for particulates. The silicone rubber seal prevents the leakage of electrolyte. The PTFE frame connecting with silicone rubber tubes is the gateway for electrolytes pumping into and out of the cell. The separator keeps the two electrodes apart to prevent electrical short-circuit while allowing the transport of ions during charge/discharge processes.

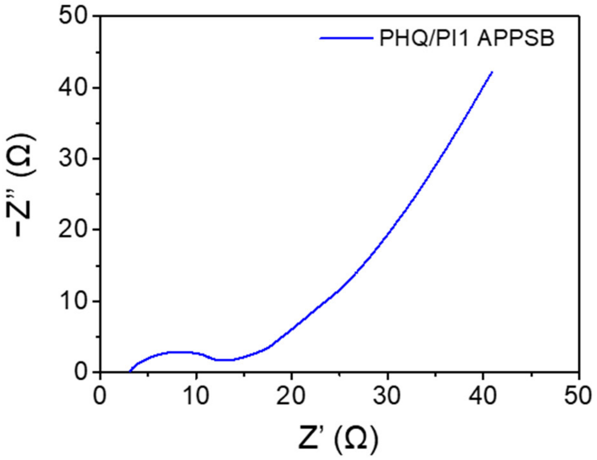

Supplementary Figure 12. EIS curve of PHQ/PI1 APPSBs.

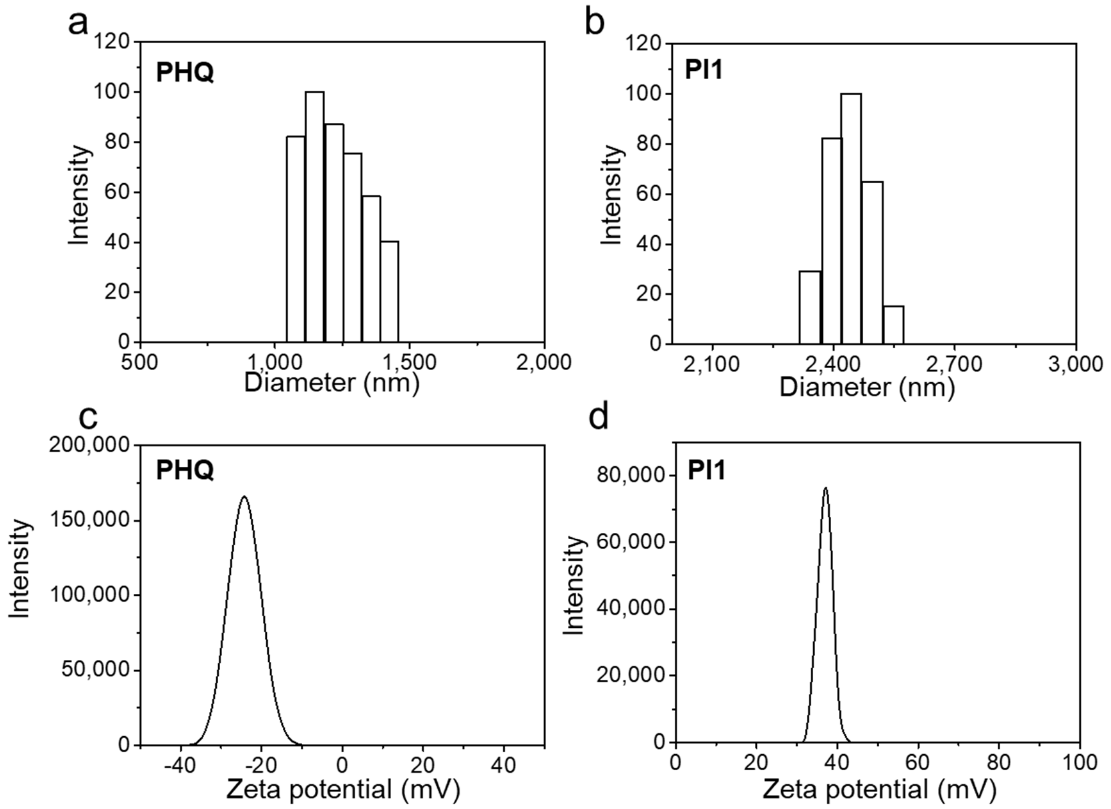

Supplementary Figure 13. Size distribution and Zeta potential of polymer particulate suspensions. DLS distribution and Zeta potential of (**a**, **c**) PHQ and (**b**, **d**) PI particulate suspensions after charging/discharging cycling, respectively.

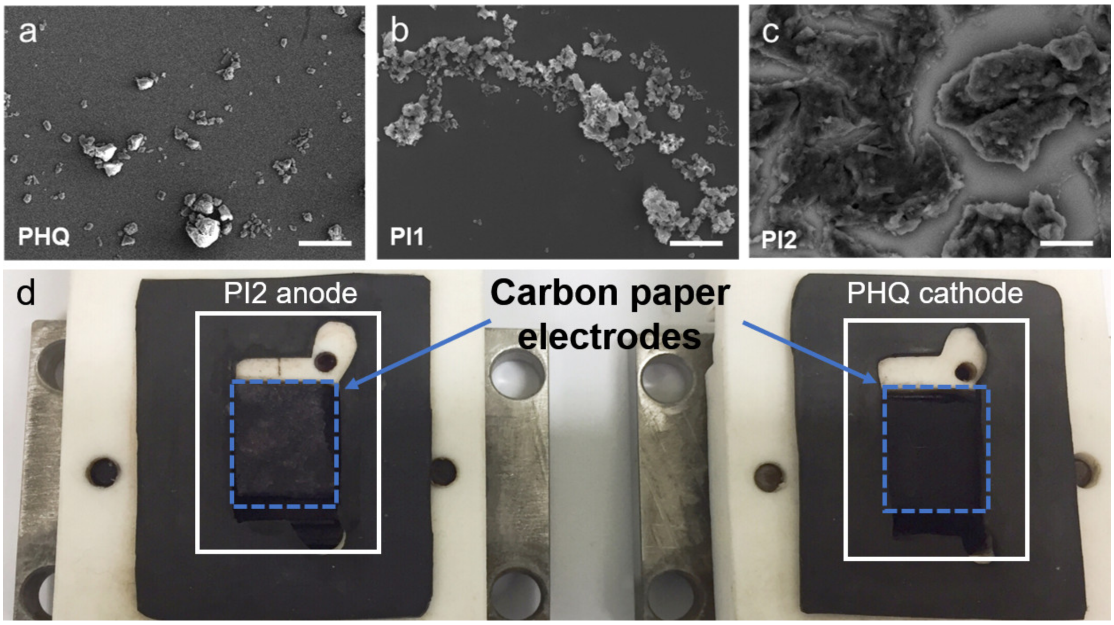

Supplementary Figure 14. Postrun analysis of polymer particulate agglomeration degrees after long-term cycling. SEM images of (**a**) PHQ, (**b**) PI1, and (**c**) PI2 particulates after after charging/discharging cycling. Scale bars, (**a**, **b**) 20 µm; (**c**) 5 µm. (d) Optical photographs of disassembled PHQ/PI2 APPSB cell after long-term cycling test.

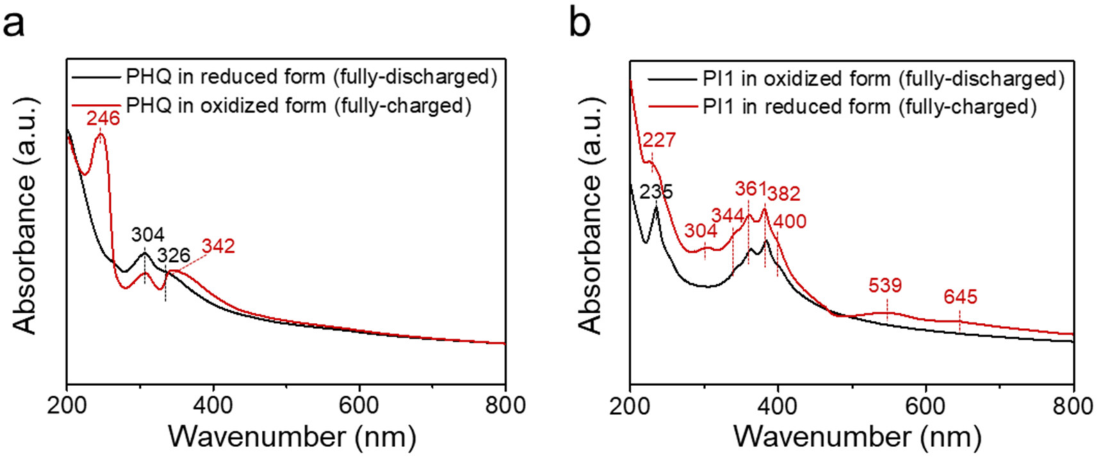

Supplementary Figure 15. UV-Vis absorption spectra of the diluted polymer particulate suspensions at fully charged and discharged states. The polymer particulate suspensions at fully-discharged state represent the reduced form of PHQ and the oxidized form of PI1. The polymer particulate suspensions at fully-charged state represent the oxidized form of PHQ and the reduced form of PI1.

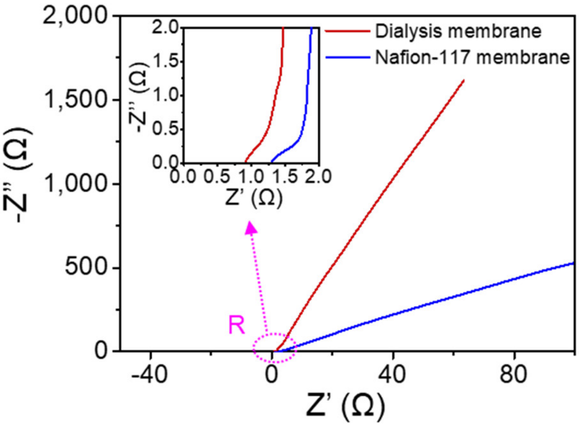

Supplementary Figure 16. EIS measurements of dialysis membrane (MWCO1,000) and Nafion-117 membrane.

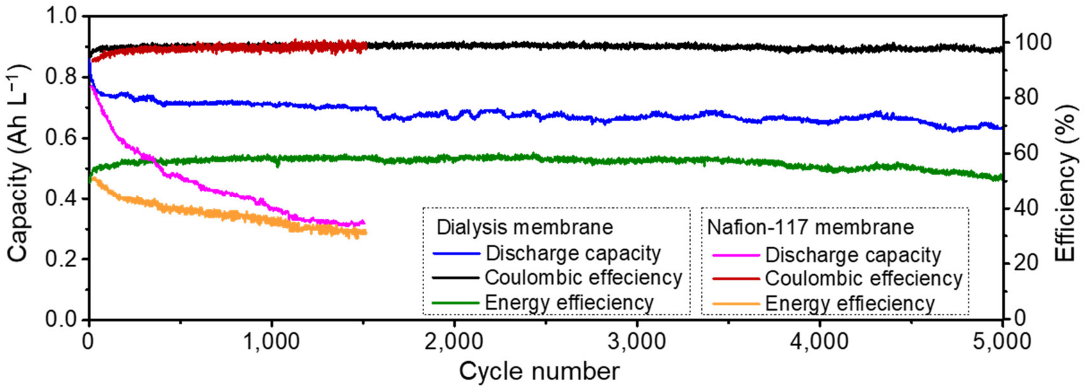

Supplementary Figure 17. Long-term cycling performance comparison of PHQ/PI1 APPSBs assembled with dialysis membrane or Nafion-117 membrane separator. The PHQ/PI1 APPSBs based on 0.1 mol L^{-1} polymer particulate suspensions were charged and discharged at the current density of 20 mA cm^{-2}, under static operation.

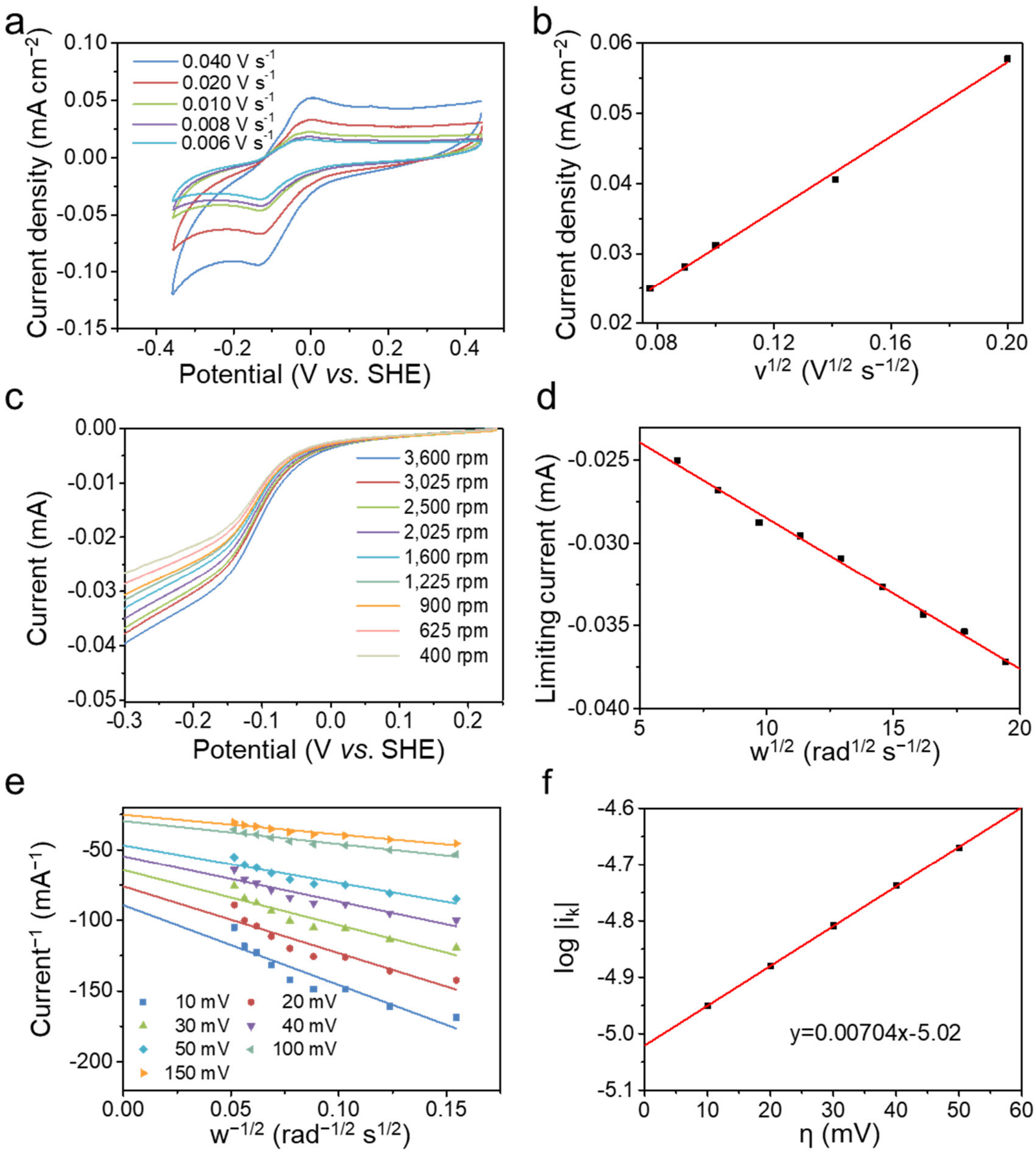

Supplementary Figure 18. Electrochemical characterizations of PI2 particulate suspension. (**a**) Cyclic voltammograms of 0.1 mol L^{-1} PI2 in 2.0 mol L^{-1} H_2SO_4 aqueous solution at different scan rates. (**b**) Reduction peak current (i) versus scan speed. (**c**) RDE measurements at rotating electrode speeds from 400 rpm to 3,600 rpm using 0.005 mol L^{-1} PI2 in 2.0 mol L^{-1} H_2SO_4 aqueous solution. (**d**) Limiting current versus the square root of rotation velocity (Levich plot). (**e**) Koutecky′–Levich plot. (**f**) Tafel plot.

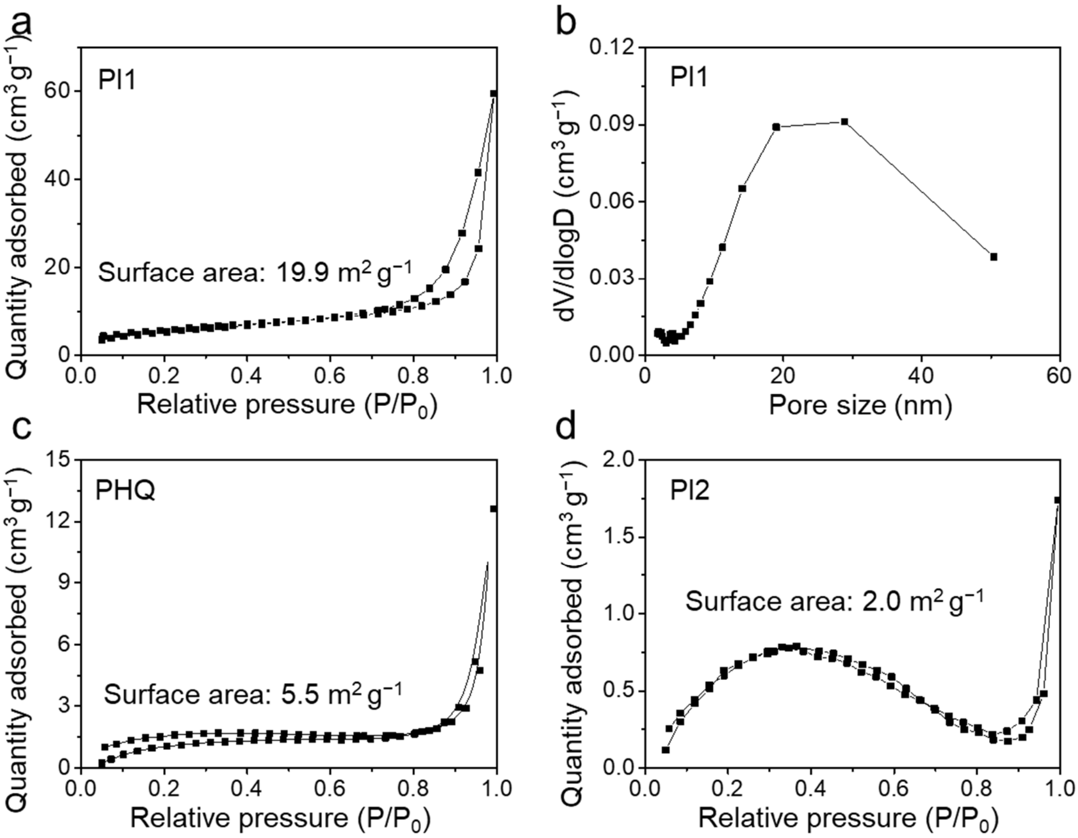

Supplementary Figure 19. Surface area and pore size characterizations. N_2 adsorption−desorption isotherms of (**a**) PI1, (**c**) PHQ and (**d**) PI2 particulates, respectively. (**b**) Pore size distribution of PI1 particulates.

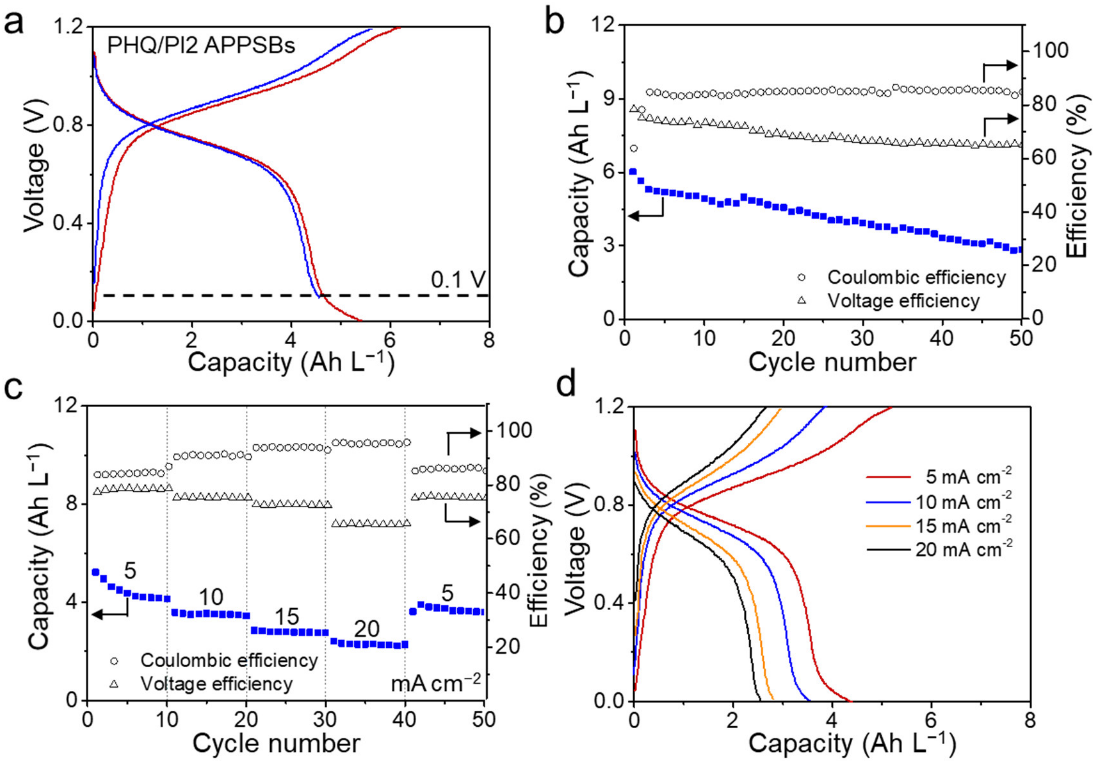

Supplementary Figure 20. Performances of PHQ/PI2 APPSBs assembled with dialysis membrane separators. (**a**) Representative charge/discharge curves measured at 5 mA cm^{-2}. (**b**) Cycling stability measured at 5 mA cm^{-2}. (**c**) Rate performance, Coulombic efficiencies and voltage efficiencies measured between 5–20 mA cm^{-2}. (**d**) Representative charge/discharge curves measured at different current densities.

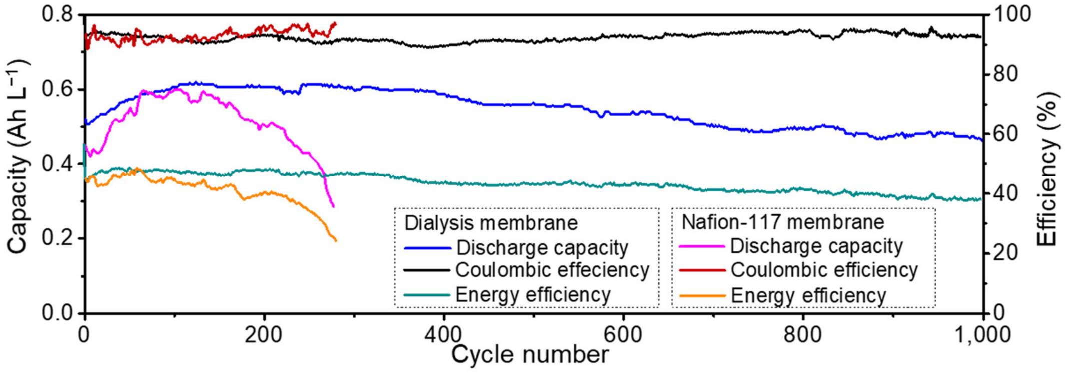

Supplementary Figure 21. Long-term cycling performance comparison of PHQ/PI2 APPSBs with dialysis membrane or Nafion-117 membrane separator. The PHQ/PI2 APPSBs based on 0.1 mol L^{-1} polymer particulate suspensions were charged and discharged at the current density of 20 mA cm^{-2}, under static operation.

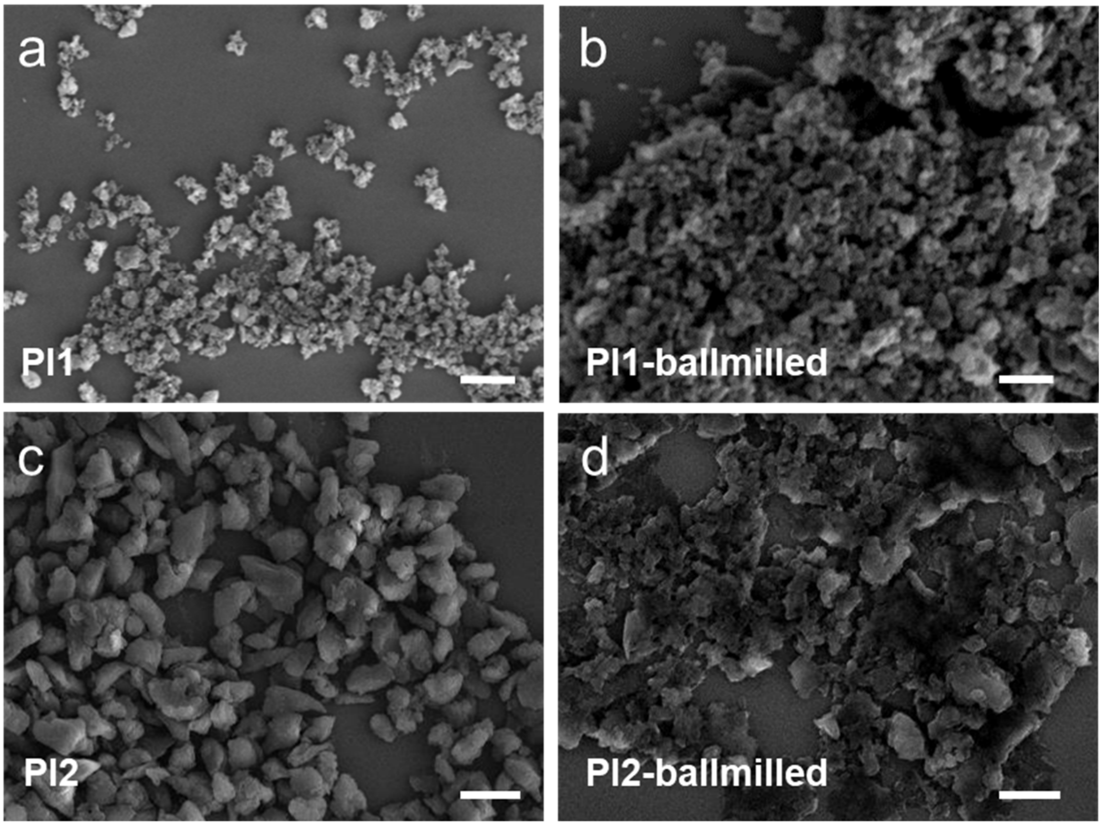

Supplementary Figure 22. SEM characterizations of PI particulates with different sizes. SEM images of (**a**) PI1, (**b**) PI1-ballmilled, (**c**) PI2 and (**d**) PI2-ballmilled, respectively. Scale bars, (**a**, **c**) 5 μm; (**b**, **d**) 1 μm.

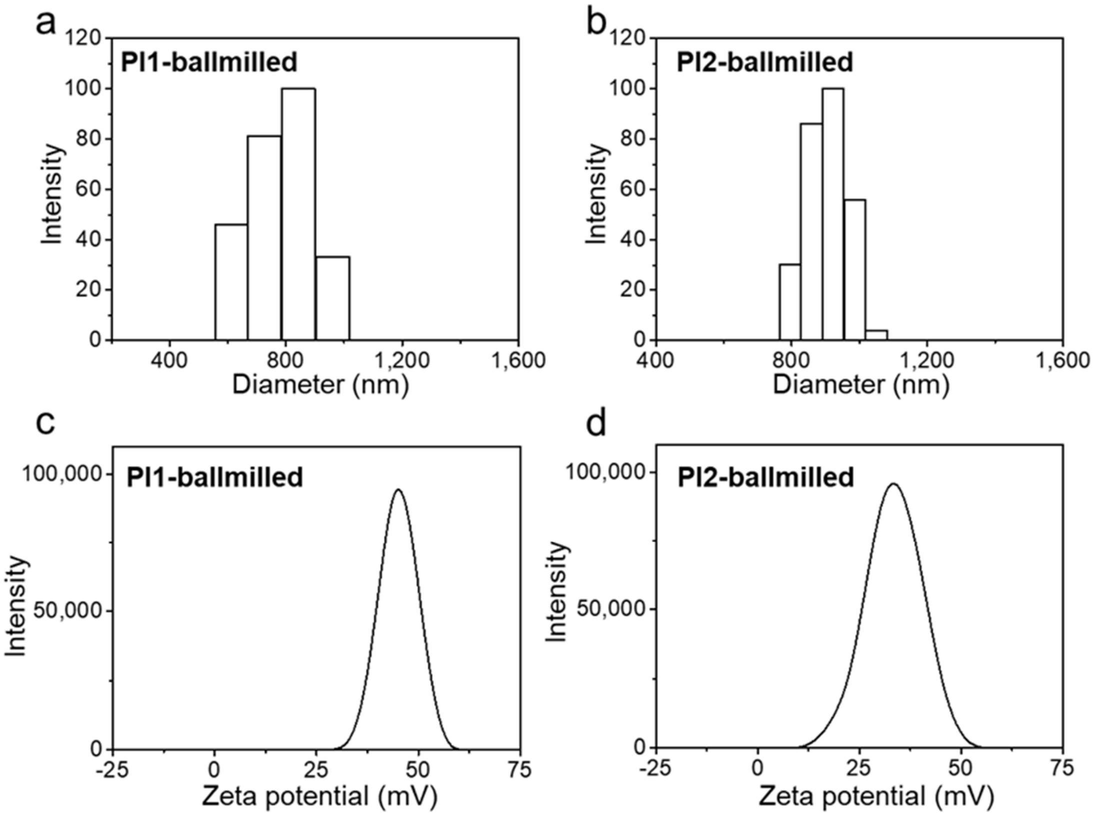

Supplementary Figure 23. Size distribution and dispersion stability of ballmilled PI particulate suspensions. DLS diameter distributions of (**a**) PI1-ballmilled and (**b**) PI2-ballmilled. Zeta potential curves of (**c**) PI1-ballmilled and (**d**) PI2-ballmilled.

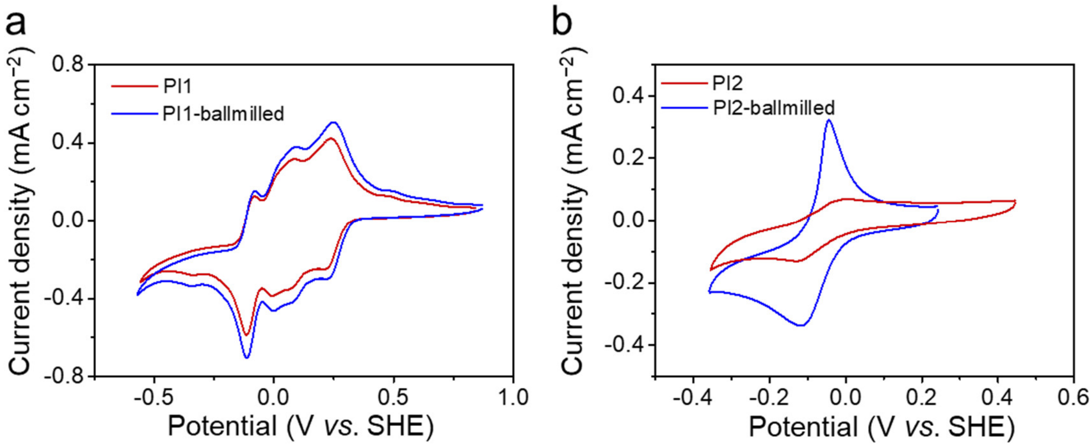

Supplementary Figure 24. CV analysis of PI particulate suspensions with different particle sizes. CV curves of 0.005 mol L^{-1} PI particulate suspensions with different sizes in 2.0 mol L^{-1} H_2SO_4 aqueous solution: (**a**) PI1 and PI1-ballmilled, (**b**) PI2 and PI2-ballmilled, respectively. The scan rate is 0.025 V s^{-1}.

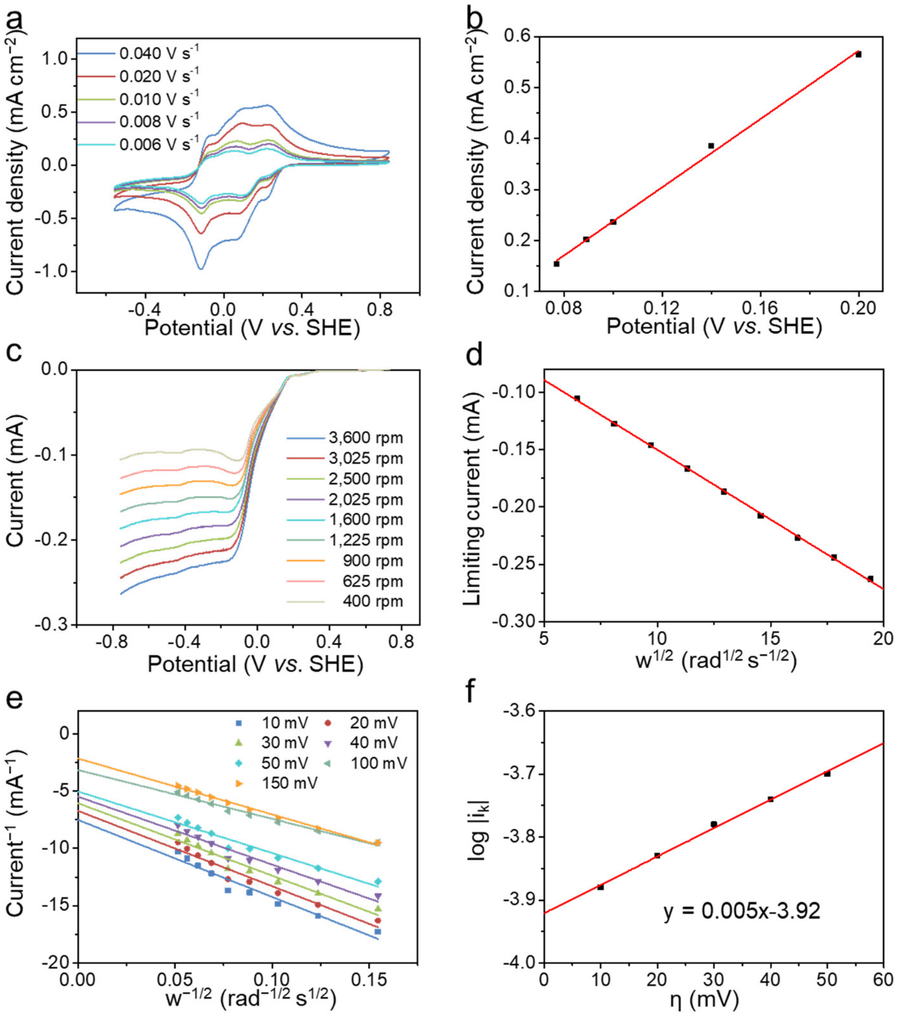

Supplementary Figure 25. Electrochemical characterizations of PI1-ballmilled. (**a**) Cyclic voltammograms of 0.1 mol L^{-1} PI1-ballmilled in 2.0 mol L^{-1} H_2SO_4 aqueous solution at different scan rates. (**b**) Oxidation peak current (i) versus scan speed. (**c**) RDE measurements at rotating electrode speeds from 400 rpm to 3,600 rpm using 0.005 mol L^{-1} PI1-ballmilled in 2.0 mol L^{-1} H_2SO_4 aqueous solution. (**d**) Limiting current versus the square root of rotation velocity (Levich plot). (**e**) Koutecky′–Levich plot. (**f**) Tafel plot.

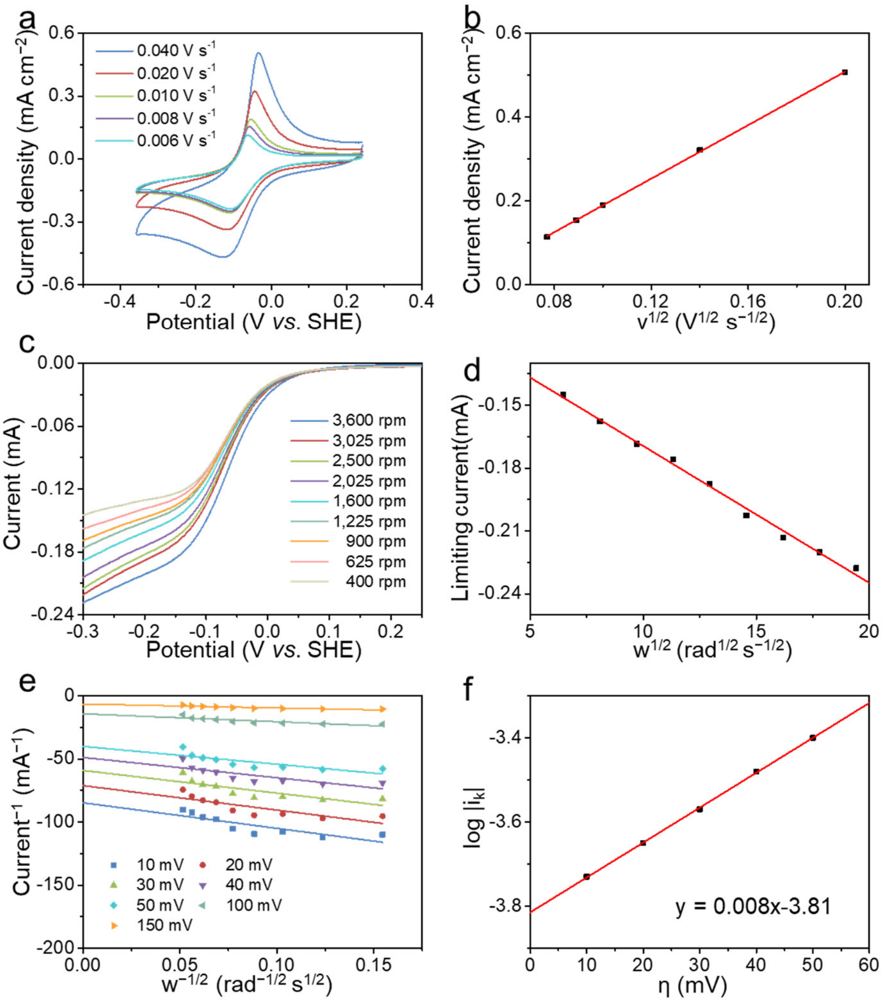

Supplementary Figure 26. Electrochemical characterizations of PI2-ballmilled. (**a**) Cyclic voltammograms of 0.1 mol L^{-1} PI2-ballmilled in 2.0 mol L^{-1} H_2SO_4 aqueous solution at different scan rates. (**b**) Oxidation peak current (i) versus scan speed. (**c**) RDE measurements at rotating electrode speeds from 400 rpm to 3,600 rpm using 0.005 mol L^{-1} PI2-ballmilled in 2.0 mol L^{-1} H_2SO_4 aqueous solution. (**d**) Limiting current versus the square root of rotation velocity (Levich plot). (**e**) Koutecky′–Levich plot. (**f**) Tafel plot.

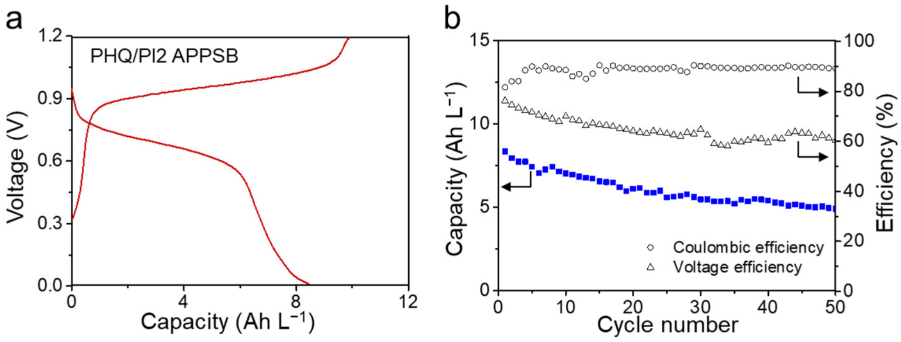

Supplementary Figure 27. Performances of PHQ/PI2-ballmilled APPSBs. (a) Representative charge/discharge curves of PHQ/PI2-ballmilled APPSBs at 5 mA cm^{-2}. (**b**) Stability test of PHQ/PI2-ballmilled APPSBs at the current density of 5 mA cm^{-2}.

Supplementary Tables

Supplementary Table 1. Performance comparisons of the APPSBs in this work with other representative vanadium-based and organic-based RFBs.

Sources	Electrolyte	Redox-active material	Electron-transfer rate constant k_0 ($cm\ s^{-1}$)	Concentration ($mol\ L^{-1}$)	Current density ($mA\ cm^{-2}$)	Energy density ($Wh\ L^{-1}$)	Cycle number	Capacity retention
**This work**	H_2SO_4 (aq)	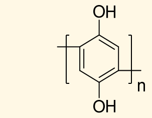	6.72×10^{-4}	0.1, 1.0	5-20	2-6	300<br>5,000 (non-flow cell)	70%<br>74%
		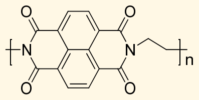	2.31×10^{-3}	0.1, 1.0				
		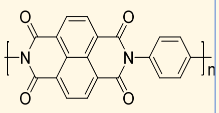	1.01×10^{-4}	0.1, 1.0				
**Ref. 1—3**	H_2SO_4 (aq)	V^{3+}/V^{2+}	5.3×10^{-4}	2	200	10-20	5,000	—
		VO^{2+}/VO_2^{+}	2.8×10^{-6}					
**Ref. 4**	NaCl (aq)	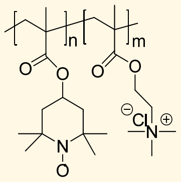	4.5×10^{-4}	0.07, 0.37	20	3.6	10,000 (non-flow cell)	80%
		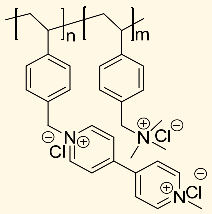	9×10^{-4}	0.15, 0.37	40	10	100	67%
**Ref. 5**	KCl (aq)	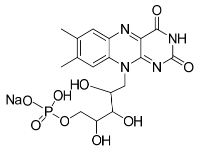	5.3×10^{-3}	0.24 (Maximum: 1.5)	80	4.8	100	99%
**Ref. 6**	KOH (aq)	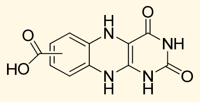	1.2×10^{-5}	0.5 (maximum: 2)	100	12	400	91%
**Ref. 7**	NaCl (aq)	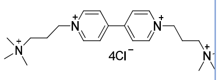	2.2×10^{-2}	0.75, 1.3	50	5	500	97%
		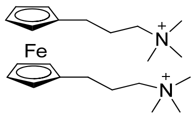	1.4×10^{-2}	1, 1.3	50	9.6	250	99%

**Ref. 8**	0.1 mol L^{-1} $TBAPF_6$ in acetonitrile	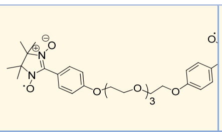	1.42×10^{-2}	0.1 (maximum 3.8)	1	4.1	20	60%
**Ref. 9**	1.0 mol L^{-1} LiTFSI in DME	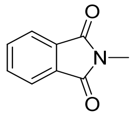	2.46×10^{-3}	0.3, 0.3	35	6.5	50	95%
		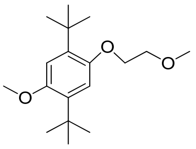	1.35×10^{-2}	0.1, 0.1	10	2.3	100	85%
**Ref. 10**	NaCl (aq)	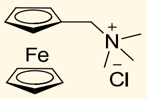	3.66×10^{-5}	0.5, 0.5 (maximum: 3)	60	9	700	91%
		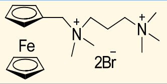	4.60×10^{-6}	0.7, 0.7 (maximum: 2)	60	9.9	500	81%
**Ref. 11**	NaCl (aq)	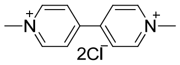	2.8×10^{-4}	0.5, 0.5 (maximum:3) 0.5	60	7.5	100	89%
		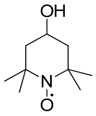	2.6×10^{-4}					
**Ref. 12**	H_2SO_4 (aq)	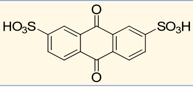	7.2×10^{-3}	0.1, 1	200	1	15	99%
		Br_2	—	0.5, 0.5	500	16	10	92%
**Ref. 13**	0.1 mol L^{-1} $LiBF_4$ in acetonitrile	Viologen-based colloids	1.5×10^{-2}	0.01 (maximum: 2)	0.043	0.05	50	100%
		Ferrocene-based colloids	—	0.01				

Supplementary Table 2. Physicochemical and electrochemical properties of polymer particulates with different particle sizes.

Materials	Average size (μm)		Zeta potential (mV)	Oxidation peak current (mA cm^{-2})	Polarization (mV)	Diffusion Coefficient (10^{-7} cm^2 s^{-1})	Electron transfer rate constant (10^{-3} cm s^{-1})	Viscosity (mPa s)
	SEM	DLS						
**PI1**	2.5	2.7	48.6	0.42	38	1.7	2.31	3.1
**PI1-ballmilled**	0.9	0.8	47.8	0.50	30	3.4	1.33	5.2
**PI2**	5.6	5.6	10.6	0.07	91	0.7	0.10	2.3
**PI2-ballmilled**	0.9	0.9	34.3	0.32	57	1.3	0.81	4.7

Supplementary References

1. Yamamura, T., Watanabe, N., Yano, T., Shiokawa, Y. Electron-transfer kinetics of Np^{3+}/Np^{4+}, NpO_2^{+}/NpO_2^{2+}, V^{2+}/V^{3+}, and VO^{2+}/VO_2^{+} at carbon electrodes. *J. Electrochem. Soc.* **152,** A830–A836 (2005).
2. Yang, Z. G. et al. Electrochemical energy storage for green grid. *Chem. Rev.* **111,** 3577–3613 (2011).
3. Lu, W. J., Shi, D. Q., Zhang, H. M. & Li, X. F. Highly selective core-shell structural membrane with cage-shaped pores for flow battery. Energy Storage Materials, **17,** 325–333 (2019).
4. Janoschka, T. et al. An aqueous, polymer-based redox-flow battery using non-corrosive, safe, and low-cost materials. *Nature* **527,** 78–81 (2015).
5. Orita, A., Verde, M. G., Sakai, M. & Meng, Y. S. A biomimetic redox flow battery based on flavin mononucleotide. *Nat. Commun.* **7,** 13230 (2016).
6. Lin, K. X. et al. A redox-flow battery with an alloxazine-based organic electrolyte. *Nat. Energy* **1,** 16102 (2016).
7. Beh, E. S. et al. A neutral pH aqueous organic–organometallic redox flow battery with extremely high capacity retention. *ACS Energy Lett.* **2,** 639–644 (2017).
8. Hagemann, T. et al. A bipolar nitronyl nitroxide small molecule for an all-organic symmetric redox-flow battery. *NPG Asia Mater.* **9,** e340 (2017).
9. Wei, X. et al. A high-current, stable nonaqueous organic redox flow battery. *ACS Energy Lett.* **1,** 705–711 (2016).
10. Hu, B., DeBruler, C., Rhodes, Z. & Liu, T. L. Long-cycling aqueous organic redox flow battery (AORFB) toward sustainable and safe energy storage. *J. Am. Chem. Soc.* **139,** 1207–1214 (2017).
11. Liu, T., Wei, X., Nie, Z., Sprenkle, V. & Wang, W. A total organic aqueous redox flow battery employing a low cost and sustainable methyl viologen anolyte and 4-HO-TEMPO catholyte. *Adv. Energy Mater.* **6,** 1501449 (2016).
12. Huskinson, B. et al. A metal-free organic-inorganic aqueous flow battery. *Nature* **505,** 195-198 (2014).
13. Montoto, E. C. et al. Redox active colloids as discrete energy storage carriers. *J. Am. Chem. Soc.* **138,** 13230–13237 (2016).
